# The association between women’s empowerment in agriculture and child stunting in Malawi

**DOI:** 10.1038/s41598-026-40495-6

**Published:** 2026-02-22

**Authors:** Eileen Bogweh Nchanji, Kelvin Kariuki Kamunye, Odhiambo Collins Ageyo, Yvonne Munyangeri, Mercy Mutua, Cosmas Kweyu Lutomia

**Affiliations:** 1 International Centre for Tropical Agriculture, Nairobi, Kenya; 2 Nairobi, Kenya; 3 International Centre for Tropical Agriculture, Kigali, Rwanda

**Keywords:** Women empowerment, Child stunting, Malawi, Pro-WEAI, Maternal education, Agriculture, Child nutrition, Logistic regression, Health care, Nutrition, Environmental social sciences, Socioeconomic scenarios

## Abstract

Although empowering women in agriculture is identified as a potential pathway for addressing high prevalence of malnutrition in Malawi, evidence on the association between empowerment and child nutrition remains limited. The study assessed the association between women’s empowerment and child stunting in three districts of Malawi. Data were collected using a case-control design from 847 children aged 6–59 months and their mothers. Empowerment was measured using the project-level Women Empowerment in Agriculture Index, while data on child stunting were collected using anthropometric measurements. Logistic regression results showed that adequacy in self-efficacy was associated with a 1.7% point higher probability of stunting, while ownership of land and other assets reduced probability of stunting by 14.7% points. Mother’s marital status and schooling were associated with 15.9 and 1.3 lower percentage points of stunting, while child’s age increased percentage points of stunting by 0.7. The findings suggest the need for women’s empowerment programs to bundle nutrition-sensitive interventions and education to contribute to improved child nutrition. Actionable gender components such as asset security, workload management, and decision-spaces are also recommended.

**Supplementary Information:**

The online version contains supplementary material available at 10.1038/s41598-026-40495-6.

## Introduction

Adequate nutrition, defined as to the intake of sufficient energy, macronutrients, and micronutrients to support optimal growth, development, and physiological function, is important for the well-being of children, offering multifaceted benefits that enable children to achieve optimal growth and developmental milestones^1^. For example, adequate nutrition supports the physical, cognitive, emotional, and social development of children under 5 years old. Thus, ensuring adequate nutrition for children transcends immediate growth and development milestones, providing a foundation for healthier generations^2^.

The triple burden of malnutrition, encompassing stunting, wasting, and overweight, is a threat to children’s well-being. In 2022, 148.1 million, equivalent to 22.3 per cent of children under age 5 globally, were stunted^3^. Out of these, 43 per cent lived in Africa^3^. Stunting results from inadequate nutrition in-utero and early childhood periods. Children experiencing stunting might never achieve their maximum height potential and cognitive developmental milestones. Stunting has lasting effects into adulthood, including reduced cognitive abilities, susceptibility to infections and illnesses, higher risk of reproductive health issues, and reduced physical and economic capacity^4^. Therefore, the high prevalence of malnutrition, especially stunting, is a major nutritional concern impacting health and development in sub-Saharan Africa.

In Malawi, Vitamin A deficiency affects approximately 60% of children under five, while iron deficiency anaemia is prevalent among over 30% of women of reproductive age and more than 60% of children aged 6–59 months^5–7^. These deficiencies impair immune function, cognitive development, and physical growth, contributing to the persistence of stunting despite improvements in food availability. Zinc deficiency is also widespread, driven by low dietary diversity and reliance on maize-based staples^8^.

In Malawi, child malnutrition remains a serious challenge. It is associated with 23% of mortality for children under the age of five years and an annual gross domestic product loss of about 10.3%^9,10^. Prevalence of stunting, that is, the percentage of children under five years who were stunted, was 34% in 2022, down from 62% in 1998 and 44% in 2012^2,11,12^. Despite some improvements in the last decade, the stunting rate remains high, necessitating alternative interventions to curb the malnutrition challenge.

Traditional approaches to addressing malnutrition in low-income countries have mostly focused on improving the production, availability, and affordability of food. However, it is important to recognize that poor nutrition outcomes are not solely due to resource constraints, but also due to other pathways related to food choice behaviours and eating practices, such as cultural preferences, knowledge, and social norms^13^. Consequently, there has been a growing interest in understanding the association between women’s empowerment in agriculture and nutrition outcomes, especially among children under five years of age^14–17^. Women’s agency in decision-making and control over resources are linked to maternal and child nutrition outcomes^18,19^. Empowered women are reported to be more likely to allocate resources towards children’s nutritional wellbeing compared to less empowered women^20,21^. This highlights the need to leverage women’s empowerment in agriculture in the design of interventions that aim to address child malnutrition^13,22^.

Empirical work linking women’s empowerment to nutrition in Malawi remains limited despite sustained nutrition programming aimed at addressing malnutrition in the country. A recent pro-WEAI^23^ and market inclusion study in Malawi showed that training and extension are associated with higher empowerment. Still, it did not directly link empowerment to nutrition outcomes at the household level, especially among children under 5^24^. This indicates that while progress has been made in understanding what drives empowerment, less is known about how such empowerment translates into improved nutrition outcomes in contexts where stunting persists. Besides, pro-WEAI has been increasingly used to assess the impact of agricultural interventions on women’s empowerment and nutrition outcomes in various global contexts, but its application in Malawi remains limited. This underscores the need for further research that integrates multidimensional empowerment metrics—such as pro-WEAI—into nutrition-sensitive programming. We address this gap by applying the pro-WEAI to mothers of children aged 6–59 months in three ecologically distinct districts to estimate the association between specific women’s empowerment in agriculture indicators and child stunting. Our study contributes to this discourse by highlighting empowerment-nutrition linkages, and by identifying areas where pro-WEAI could enhance future evaluations and interventions.

We acknowledge that maternal health during pregnancy and conditions at birth are important factors influencing nutrition outcomes among children under 5^25,26^. However, given high prevalence of stunting in Malawi and its policy salience (e.g., National Multisector Nutrition Policy and Strategic Plan (2025–2030) and the Malawi 2063 (MW2063)), we use stunting as proxy for child nutrition rather than other nutrition outcomes, such as wasting, that are more sensitive to short-term changes in the food and health environment. Child stunting is defined as a binary variable indicating whether a child’s height-for-age Z-score (HAZ) is more than two standard deviations below the WHO reference population median^27^. While the study did not directly collect maternal nutrition during pregnancy or birth conditions as potential determinants of stunting as identified in recent longitudinal evidence^25,26^, it incorporated maternal education, marital status, and occupation as explanatory variables to reflect structural and social dimensions that shape the underlying pathway from maternal health and caregiving capacity to child nutrition.

## Conceptual framework

This study is guided by a conceptual framework that links women’s empowerment to child nutritional outcomes through multiple, interrelated pathways. We hypothesized that women’s adequacy in empowerment indicators shapes household resource allocation, caregiving practices, and access to services, which translates into child nutrition. Decision-making power over agricultural production, access to and control over land and income, participation in farmer groups, time allocation between productive and reproductive roles, and mobility in accessing markets and services are some of the empowerment domains in the pro-WEAI likely to influence child nutrition through intermediate pathways such as improved household food availability and diversity, increased income and expenditure on child needs, enhanced maternal caregiving, and greater utilization of health services. The framework hypothesizes that empowered women are more likely to allocate resources toward food and health expenditures^28,29^. We expect an inverse association between empowerment – as measured by the adequacy in pro-WEAI indicators – and child stunting because empowered women are likely to adopt improved feeding and hygiene practices^30–32^. These intermediate pathways influence immediate child dietary intake and reduce exposure to morbidity, which together determine child growth outcomes, such as height-for-age Z-scores and prevalence of stunting within households. However, the framework also acknowledged the influence of maternal and child characteristics. We hypothesized that marital status, age, and education level of the mother and age and sex of the child influence the association between empowerment and child nutrition^30^.

## Methods

### Study area

The study was conducted in three districts of Malawi: Dedza, Balaka and Mzimba South. Dedza is one of the nine districts in the Central region of Malawi, while Balaka and Mzimba districts are in the southern and northern regions, respectively. Each targeted district represents a distinct ecological zone in Malawi. Dedza and Balaka districts are characterized by subtropical highland climatic conditions. Wet and dry seasons occur between November and April and May and October, respectively. Mzimba district is characterized by moderate to high rainfall. The diverse agro-ecological conditions support the production of maize, beans, and groundnuts across the three districts. Other crops that support rural economy are potatoes and sweet potatoes in Dedza, millet and sorghum in Balaka, and tobacco, cassava, and sweet potatoes in Mzimba South.

From a demographic and socioeconomic context, while Balaka is a plural, with the population composed of various ethnic groups, Dedza and Mzimba districts are predominantly one ethic group. Women, who constitute over 50% of the population, play crucial roles in agriculture and household food security. Both Mzimba and Dedza districts have high proportions of children under the age of five, making child nutrition a critical issue. Despite the crucial role they play in rural livelihoods, women in the three districts often face limited access to resources and decision-making power that undermines their contribution to health and nutrition. Stunting in Dedza (43%) and Mzimba (39%) is above the national prevalence rate (34%), while 33% of children in Balaka are stunted^33^.

International development agencies have implemented several interventions recently aimed at contributing to enhanced health and nutrition at individual and household levels. Examples of these projects are Afikepo Nutrition Programme (2017–2022), Improving Food Security and Nutrition Policies and Programme Outreach (IFSN) (2011–2015) by FAO, Malawi Nutrition and HIV/AIDS, Maziko Integrated Maternal and Child Grant project, GIZ’s Food and Nutrition Security Programme (FNSP), and the Feed the Future Malawi. For example, Mzimba district was among the initial target districts of the Afikepo’s Nutrition Programme and the Nutrition Programme and IFSN.Specifically, Maziko project in Balaka district aimed to improve child and maternal nutrition through financial empowerment via unconditional cash transfers, alongside social and behaviour change communication (SBCC) campaigns that actively involved men and addressed structural barriers like gender inequality and limited-service access^34^. It also involved behavioural change outreach interventions. The midterm evaluation of Maziko reported increased men’s participation in maternal and child health and improved women’s decision-making on nutrition and healthcare. The GIZ’s FNSP also used SBCC with an emphasis on gender-transformative programming aimed at enhancing food and nutrition at the household level in Dedza district^35^. Results indicated increased joint decision-making on food purchases and positive changes at the household level, which contributed to improved maternal dietary diversity. Thus, results from previous and ongoing interventions highlight the potential of variations in women’s empowerment across districts that may have influenced empowerment indicators measured in this study.

### Survey design and sampling

Data were collected from households that had at least one child under 60 months in three districts of Malawi namely: Dedza, Balaka and Mzimba South. The districts were purposively selected based on a combination of factors, including stunting prevalence, agro-ecological diversity, and presence of past or ongoing nutrition-sensitive interventions. While the most recent food and nutrition data from the Demographic and Health Survey of the Government of Malawi that showed Balaka’s stunting prevalence (33%) slightly below the national average (34%), its selection was justified by its semi-arid conditions, history of food insecurity, and its inclusion in nutrition intervention programs such as the Maziko Project. Another determinant for their selection was the intensity of project activities since the three districts were major production areas for Malawi Seed Industry Development Project Phase II (MSIDP II) target crops^33^.

The sample size was determined using standard statistical procedures for estimating proportions in cross-sectional studies, assuming a 95% confidence level and 5% margin of error for an infinite population^36^. In addition to stunting, sample size determination also considered the minimum number of respondents and household requirements recommended for the computation of pro-WEAI. The Pro-WEAI survey guidelines recommend a minimum of 400 households (corresponding to at least 800 individuals – two per household)^37^. Consistent with standard practice in pro-WEAI studies^23,38,39^, the sample was designed to ensure that no more than 20% of sampled households were female-adult-only households. Female-adult only households are defined as households without a male decision maker present^40^. Additionally, a 10% buffer was included to account for potential non-response.

A multi-stage clustered sampling approach was used for household selection. The selection of the districts was based on accessibility, presence of relevant interventions, and ecological diversity. The first phase involved randomly selecting 31 enumeration areas (EA) from each of the three districts. Within each selected EA, a complete household listing was conducted to identify eligible households with at least one woman of reproductive age and a child under five years. This listing formed the sampling frame for systematic household selection. From these lists, systematic random sampling was used to select 40 households with children below 60 months within each EA for screening.

A total of 3687 children (1202, 1217, 1268 from Balaka, Dedza and Mzimba, respectively) from 3184 households were screened for height, weight and MUAC. Due to various reasons, like some children not having female caregivers and some mothers being possibly pregnant at the time of screening, not all caregivers qualified for screening. Though 3423 children were presented by their mothers for screening, only 2930 mothers were screened for height, weight and MUAC. Based on the screening data, a matched case control design, where households with at least one stunted child were matched 1:1 with those without, controlling for child age, sex and location, was applied to generate a final sample of 924. This final sample was targeted for administration of the pro-WEAI, with 847 households successfully responding to the survey. Of these households, 711 were dual-adult households – defined as households with both male and female decision makers present^40^– and 136 female-adult only households. In a dual-adult household, one respondent was targeted as a primary decision-maker and another as a secondary decision-maker. The distribution of the sample by district was 411 in Balaka, 453 in Dedza, and 499 in Mzimba South.

### Data collection

Phase two involved taking anthropometric measurements (weight, height, and middle upper arm circumference) of 3687 children (1202, 1217, 1268 from Balaka, Dedza and Mzimba, respectively) and 2930 mothers from 3184 households. Demographic characteristics of children under five and their mothers were also collected. Phase three involved a survey of 924 households derived by matching households with a stunted child to households without a stunted child, controlling for child sex, age and location. This resulted in 847 children – 422 male and 425 female – who were included in the analysis. Consequently, 847 mothers were included in the analysis.

Data were collected using women’s empowerment domains measured by the pro-WEAI. The three domains of agency – intrinsic (power within), instrumental agency (power to), and collective agency (power with) – and their respective indicators are described in Table 1.

**Table 1:** Indicators of empowerment and definitions of adequacy.

Indicator	Definition of adequacy
Intrinsic agency (power within)	
Self-efficacy	“Agree” or greater on average with self-efficacy questions: accomplishing tasks, obtaining important outcomes, confident in performing effectively on many different tasks, and performing quite well even if things are tough.
Autonomy in income	More motivated by own values than by coercion or fear of others’ disapproval
Attitudes about intimate partner violence	Woman believes a husband is not justified to hit his wife under any of 5 listed circumstances (going out without informing him, neglecting children, denying him sex, burning food while cooking, and arguing with him).
Input in productive decisions	Woman makes decisions or feels she can make decisions in all productive activities that she participates in
Instrumental agency (power to)	
Ownership of land and other assets	Woman owns land or at least 3 of the listed agricultural assets
Access to and decisions on financial services	Woman made decisions to borrow or use credit; or did not use credit but had access if she needed to; or has access to a financial account
Control over use of income	Woman has input on utilization (keep for home consumption, sale) of agricultural outputs and use of income from non-farm economic activities for all activities in which she is actively involved
Work balance	Works less than 10.5 h per day: Workload=time spent in primary activity + (1/2) time spent in childcare as a secondary activity
Visiting important locations	Visits at least 2 of 3 places (city, market, family) weekly; or visits a hospital or public village gathering at least once every month.
Collective agency (power with)	
Group membership	Woman is an active member in at least one group in the community

Influential groups are defined as those perceived by the respondent to influence community decisions or outcomes.

Source: Malapit et al. (2019).

### Data analysis

Data analysis involved calculation of two sub-indices: the Three Domains of Empowerment (3DE) and the Gender Parity Index (GPI). The 3DE measures the extent to which men and women are empowered across 10 indicators grouped into intrinsic agency (power within), instrumental agency (power to), and collective agency (power with). Each indicator is binary (1 = adequate, 0 = inadequate), and the 3DE is calculated as the proportion of indicators in which an individual is empowered, with higher values indicating greater empowerment^23^. The GPI sub-index assesses the empowerment of women relative to men within dual-adult households. It represents the proportion of women with empowerment scores equal to or greater than those of their male counterparts^23^.

Following the standard pro-WEAI methodology, each of the 10 indicators was assigned an equal weight of 1/10. These weights are predefined in the pro-WEAI guidelines and are implemented through the Stata *weai* command^41^. The cutoff score for each indicator used to calculate 3DE is also presented in Table 1. An individual is considered adequately empowered when they are adequate in 8 of the 10 pro-WEAI indicators: a threshold of 0.8^23^. Analysis of these indicators involved cross-tabulations to obtain the distribution of women who were adequate and inadequate in each indicator. The distribution of continuous (mean) and categorical (frequencies and percentages) variables was tested for systematic differences by stunting status using an independent samples t-test and a chi-square test of independence, respectively.

We employ logistic regression to estimate the association between empowerment and stunting, controlling for maternal and child characteristics, since the outcome variable (child stunting) is a binary indicator of stunting, where 1 indicates the presence of a stunted child and zero indicates the absence of a stunted child in a household. Thus, the child is the unit of analysis, while maternal characteristics are incorporated as control variables that may influence child nutrition outcomes. This model estimates the probability of child stunting based on various empowerment and maternal factors.

Let ${y_i}$denote a household with a stunted child *i* (${y_i}=1{\text{ if HAZ < - 2, 0 otherwise}}{\mathrm{.}}$We estimate a logit:

1 $${\mathrm{Pr(}}y_{i} |{\mathrm{W}}_{i} ,{\text{ M}}_{i} {\text{ D}}_{i} ) = \Lambda (\alpha + {\mathrm{W}}^{\prime } \beta {\text{ + M}}^{\prime } _{i} \gamma + {\mathrm{D}}^{\prime } _{i} \delta ),{\text{ }}\Lambda {\text{(t) = }}\frac{1}{{1 + e^{{ - t}} }}$$

Here, ${{\mathrm{W}}_i}$are pro-WEAI indicators, ${{\mathrm{M}}_i}$maternal/child characteristics, and ${{\mathrm{D}}_i}$district dummies; Balaka is the reference category.

For a continuous regressor, ${x_{ik}}$the marginal effect (ME), reported as the average ME across *i*, is:

2 $${\mathrm{ME}}_{k} {\mathrm{(}}i{\text{) = }}\frac{{\partial {\mathrm{Pr(}}y_{i} = 1)}}{{\partial x_{{ik}} }} = \lambda (\alpha + {\mathrm{X}}^{\prime } _{i} \theta )\theta _{k} ,{\text{ }}\lambda {\mathrm{(}}t) = \Lambda (t)(1 - \Lambda (t))$$

For a binary regressor${d_{ij}}$the ME is the discrete change:

3 $${\mathrm{M}}{{\mathrm{E}}_j}=\frac{1}{n}\sum {[\Lambda (\alpha +{{\mathrm{X}}_i}({d_{ij}}} =1)^{\prime}\theta ) - \Lambda (\alpha +{{\mathrm{X}}_i}({d_{ij}}=0)^{\prime}\theta )]$$

We assessed multicollinearity among variables included in logistic regression using variance inflation factors (VIF) in the full model. All VIFs were < 2.3 (range 1.02–2.21; mean VIF = 1.24), indicating low collinearity among covariates. We include Table S1, which lists the dependent variable, the pro-WEAI indicator covariates (coded as 1 = adequate, 0 = inadequate), and all control covariates included in the logit models, along with how each relates to the empowerment–nutrition framework.

## Results

### Maternal characteristics

#### Child and mother characteristics

Table 2 compares the socio-demographic characteristics of households with (cases) and without (controls) stunted children. Among stunted children, 53% were male while 47% were female, with no statistically significant difference the two proportions. The mean age of stunted children was higher than that of non-stunted children (32 vs. 26 months; *p* = 0.000). Most mothers were married (approx. 87%), with no statistically significant differences by marital status. Most mothers (86%) reported farming as their main occupation. Participation in off-farm employment was low for both mothers with and without stunted children. Educational attainment was generally low, but mothers in control households had significantly more years of schooling than those in case households (5.43 vs. 4.89; *p* = 0.009).

**Table 2:** Socio-demographic characteristics of mothers and children by stunting status (cases vs. controls).

Variable	Control (*n* = 423)	Cases (*n* = 424)	Pooled (*N* = 847)	*p*-value
Female	53.19	47.17	50.18	0.080
Average age of child in months	25.63	32.09	28.86	0.000
	(15.86)	(13.85)	(15.23)	
Average age of mother in years	28.98	28.99	28.98	0.996
	(6.97)	(6.97)	(6.97)	
Marital status of mother (% married)	88.46	85.95	87.20	0.278
Years of education	5.43	4.89	5.16	0.009
	(3.17)	(2.78)	(2.99)	
Farming as main occupation of the mother (%)	86.3	89.76	88.04	0.123
Mother engaged in off-farm occupation (%)	7.69	6.43	7.06	0.476

Standard deviations in parentheses. p < 0.01 denotes significance at the 1% level; p < 0.05 denotes significance at the 5% level; p < 0.10 denotes significance at the 10% level.

### Aggregate measure of empowerment

There was a marginal difference in Pro-WEAI scores – defined as a composite measure that reflects the combined empowerment levels (3DE) and gender parity index (GPI) – between households with (0.706) and without (0.711) a stunted child (Table 3).

Regardless of stunting status, men from both control and case households had higher 3DE index scores (0.866 for controls, 0.862 for cases) compared to women (0.697 and 0.692) (Table 3). The 3DE measures women’s empowerment across three domains – intrinsic, instrumental, and collective agency. Consequently, this suggests both a lower prevalence and lower intensity of disempowerment among men than women. The GPI – which measures gender equality within dual-adult households – was 0.832 for controls and 0.827 for cases, indicating marginal differences by stunting status. However, about 60% of dual-adult control households and 59% of households with stunted children lacked gender parity, with mean empowerment gaps of 0.278 in controls and 0.293 in cases (Table 3).

**Table 3:** Aggregate pro-WEAI indicators among women and men in control and case households.

	Control		Cases	
	Women	Men	Women	Men
Pro-WEAI	0.711		0.706	
3DE Index	0.697	0.866	0.692	0.862
Gender Parity Index (GPI)	0.832		0.827	
% Not achieving empowerment (H)	72.813	37.200	70.283	38.306
Mean disempowerment score (A)*	0.416	0.359	0.438	0.361
% Without gender parity (HGPI)	60.4		59.274	
Mean empowerment gap (IGPI)	0.278		0.293	
Number of dual households	250		248	
Number of observations	423	250	424	248

10 indicators calculated; * Refers to the mean disempowerment score among only women/men who are disempowered; 5/3DE = 1 - (H*A); GPI = 1 - (HGPI*IGPI).

Over 70% of women, compared to 37–38% of men, did not achieve empowerment (Table 3). This is further reinforced by slightly higher disempowerment scores of 0.416 among households without stunted children and 0.438 among those with stunted children compared to men (0.359 and 0.361, respectively).

### Domain-level indicators

Table 4 compares adequacies in pro-WEAI indicators between women with and without stunted children. The indicators are organized into their respective empowerment domains. Land ownership of land and assets (89.8%) and input in productive decisions (86%) were indicators in which women had relatively the highest adequacy levels. In contrast, women had lower adequacy levels in two instrumental agency indicators – time use (19.8%) and access to and decisions on credit (45/7%) – and two intrinsic agency indicators – self-efficacy (53.4%) and attitudes about IPV (52.5%). Only two of the ten indicators were statistically significant between the two groups of households. Self-efficacy was marginally higher among women from households with stunted children (56.4%) than those from control households (50.4%) (*p* = 0.079). In contrast, ownership of land and other assets was significantly higher among control households (92.2%) than case households (87.5%) (*p* = 0.024). Other indicators, intrinsic, instrumental, and group membership indicators, showed no significant differences by stunting status.

**Table 4:** Women’s adequacy in Pro-WEAI indicators by child stunting status.

Indicator		Cases (*n* = 424)	Control (*n* = 423)	Diff.	Total (*n* = 847)
*Intrinsic agency*					
Autonomy over income		68.87	70.21	−1.34	69.54
Self-efficacy		56.37	50.35	6.02*	53.36
Attitudes about domestic violence		51.89	53.19	−1.30	52.54
*Instrumental agency*					
Input in productive decisions		84.43	87.47	−3.04	85.95
Ownership of land and other assets		87.5	92.2	−4.70**	89.85
Access to and decisions on financial services		44.81	46.57	−1.76	45.69
Control over use of income		79.95	80.85	−0.90	80.4
Work balance		20.52	19.15	1.37	19.83
Visiting important locations		82.78	85.58	−2.80	84.18
*Collective agency*					
Group membership		63.68	67.61	−3.93	65.64

*, ** denote p < 0.1 and p < 0.05 respectively.

Table 5 presents the contributions of intrinsic, instrumental, and collective agency indicators to disempowerment, disaggregated by gender and stunting status. Among women in both groups, work balance, access to credit, and self-efficacy emerged as the top contributors to disempowerment. Work balance accounted for 18% (controls) and 17% (cases) of disempowerment, while credit access contributed over 13% in both groups. Credit access, group membership, and visiting important locations were significant drivers of disempowerment among men, regardless of their stunting status.

**Table 5:** Relative contribution (%) of each pro-WEAI indicator to disempowerment among women and men in control and case households.

	Controls		Cases	
	Women	Men	Women	Men
Work balance	17.85	7.59	16.59	5.63
Access to and decisions on credit	14.30	13.71	13.78	12.82
Self-efficacy	13.18	9.07	11.10	7.77
Attitudes about intimate partner violence	11.44	8.23	10.97	6.80
Group membership	8.86	11.39	9.23	10.68
Autonomy in income	8.30	7.17	8.16	7.96
Control over use of income	5.58	1.69	5.35	2.14
Visiting important locations	4.11	7.38	4.41	8.74
Input in productive decisions	3.63	1.48	4.41	1.94
Ownership of land and other assets	2.02	2.74	3.28	2.14

10 indicators calculated; The relative contribution of each indicator to disempowerment reflects how much each indicator contributes to the disempowerment index (1–5/3DE) for women and men in the sample.

### Associations between individual characteristics and stunting

**Fig. 1:**
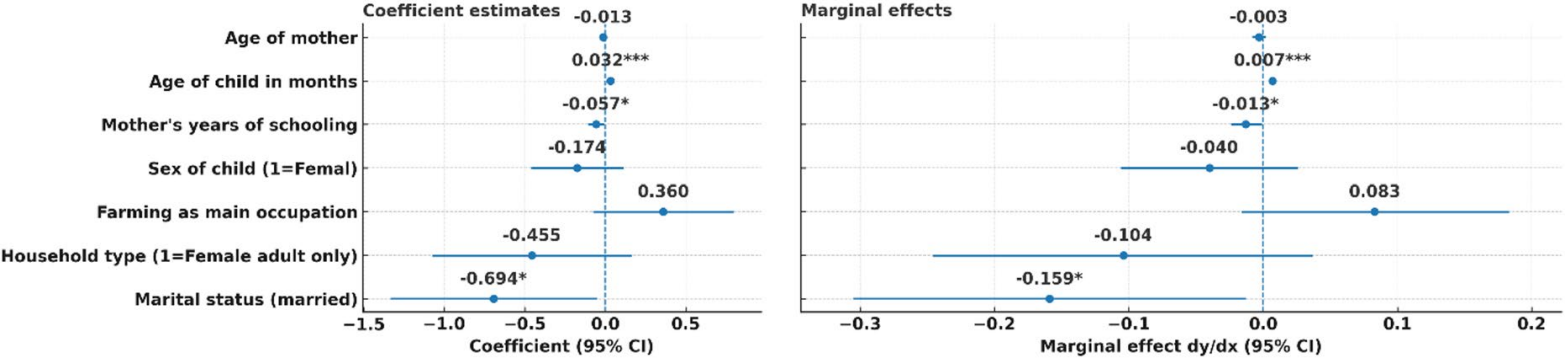
*N* = 847; Coefficients indicate the direction and strength of associations in log-odds, while marginal effects show the change in probability of stunting linked to each variable. Robust standard errors were applied to ensure reliable inference. The associations between marital status and the number of mothers’ years of schooling and stunting were significant, *p* < 0.1. The age of the child and stunting were statistically significantly associated at *p* < 0.01.

The associations of child and maternal characteristics with stunting described in Eq. (1) are graphically shown in Fig. 1. Two mother characteristics – years of schooling and being married – were negatively and marginally associated with stunting (schooling: dy/dx = − 0.013; 95% CI: −0.024 to − 0.001; *p* = 0.000; married: dy/dx = − 0.159; 95% CI: −0.305 to − 0.013; *p* = 0.035). A child’s age was positively and statistically significantly associated with stunting (dy/dx = 0.007; 95% CI: 0.005–0.009; *p* = 0.000). Other covariates, including child sex, maternal age, household type (female-adult-only), and farming as the mother’s main occupation, were not statistically significant at 5% level. Logistic regression also controlled for district-level effects.

### Associations between empowerment and stunting

**Fig. 2:**
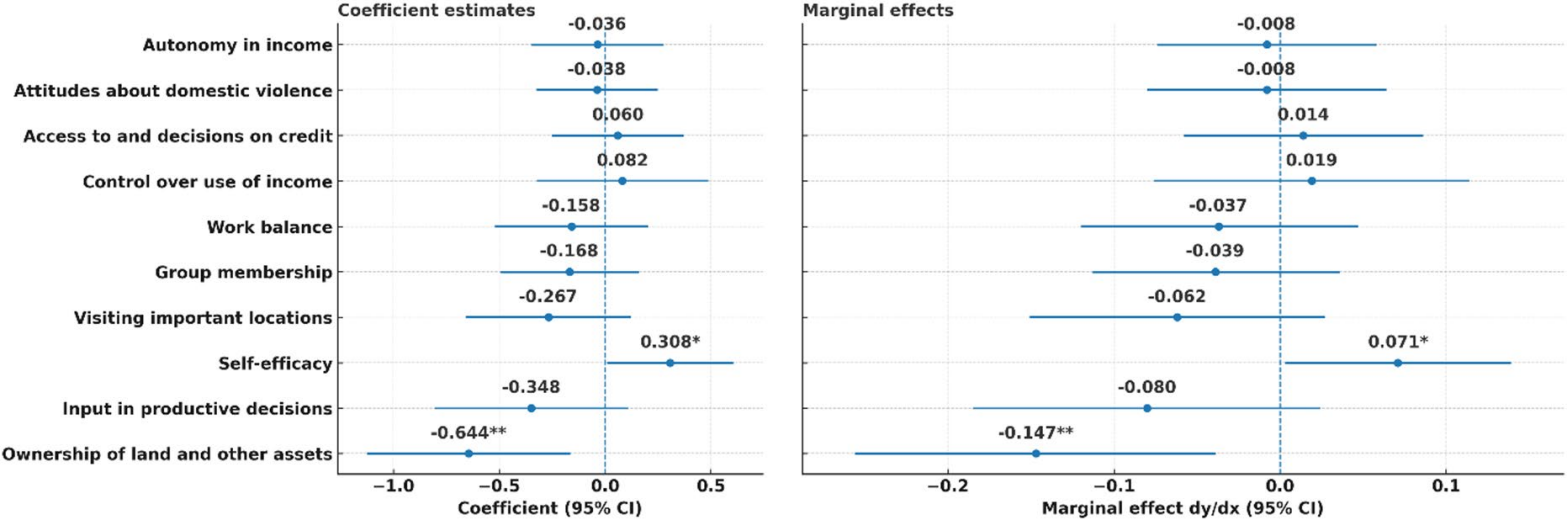
*N* = 847; Coefficients indicate the direction and strength of associations in log-odds, while marginal effects show the change in probability of stunting linked to each empowerment indicator. Robust standard errors were applied to ensure reliable inference. The association between self-efficacy and stunting were significant at *p* < 0.1. Ownership of land and other assets and stunting statistically significant at *p* < 0.05.

The associations between women’s empowerment indicators and stunting are graphically illustrated in Fig. 2. Of ten indicators, only two show statistically significant associations with stunting: ownership of land and other assets was negatively associated (dy/dx = − 0.147; 95% CI: −0.256 to − 0.039; *p* = 0.008), while self-efficacy was positively associated (dy/dx = 0.071; 95% CI: 0.003–0.138; *p* = 0.041). The remaining indicators, although directionally consistent with the expected relationship, were not statistically significant at conventional levels.

## Discussion

In this paper, we examine the association between stunting and women’s empowerment in three districts of Malawi, controlling for maternal and child characteristics. The key findings are that asset and land ownership is negatively associated with stunting, self-efficacy is positively associated, and child age is positively associated, while maternal schooling and being married are associated with lower stunting.

Pro-WEAI index score for households with and without stunted children were ideally the same (0.706 vs. 0.711). Comparable country-level benchmarks remain limited, but these scores are lower than the 0.89 pro-WEAI score for Malawi reported^24^. However, the reported scores are comparable to recent pro-WEAI applications in Uganda and Senegal that showed scores of 0.60 and 0.69, respectively^42^. Although this result suggests that the Malawi values are broadly consistent with findings in other sub-Saharan African contexts, they are slightly lower than the pro-WEAI score of 0.85 in Nepal^42^ and higher than those reported in another study in Bangladesh^43^. The cross-country variation in pro-WEAI scores likely reflects contextual differences in agricultural systems, gender norms, and policy environments.

The 3DE measures women’s empowerment across three domains – intrinsic, instrumental, and collective agency. Consequently, the finding show both a lower prevalence and lower intensity of disempowerment among men than women. Interpreted together with GPI, this result indicates that while most households approached gender parity, there remained a sizeable share without it, especially among households with stunted children. This is further reinforced by slightly higher disempowerment scores of 0.416 among children without stunting and 0.438 among those with stunted children compared to men (0.359 and 0.361, respectively). This indicates that a higher proportion of women than men was inadequate in a greater share of weighted intrinsic, instrumental, and collective agency indicators.

The positive association between self-efficacy and stunting is counterintuitive. Being adequate in self-efficacy was associated with a 1.7 pp higher probability of stunting. This result is inconsistent with studies that report an inverse relationship between self-efficacy and stunting^7,44^ and therefore merits careful and non-exhaustive interpretation. The first interpretation could be linked to mothers of growth-compromised children reporting higher self-efficacy after navigating the care-seeking process, thereby elevating measured self-efficacy conditional on stunting. In contrast, the result could be linked to time-allocation trade-offs. Women with higher self-efficacy may be engaging in activities or pursuits that demand substantial time commitments, possibly affecting their ability to allocate time to essential childcare and nutrition-related tasks like exclusive breast feeding. Either way, the unexpected association between self-efficacy and stunting is consistent with a non-significant or positive relationship between access to and decision over credit, as well as control over the use of income indicators, and low adequacy in work-life balance. This observation suggests that agency unaccompanied by care-enabling conditions may not translate into better nutrition outcomes for children under five. This caveat is stressed in the literature^45–47^.

The association between empowerment indicators and child stunting exhibited heterogeneous patterns across domains. Most empowerment indicators exhibited negative associations with stunting, indicating lower stunting probabilities when mothers are adequate in intrinsic, instrumental, and collective agencies, except for self-efficacy, access to and decision-making on credit, and control over the use of income, which were negatively signed. Ownership of assets (land and other assets) was strongly and negatively associated with child stunting. Mothers with adequate assets and land ownership had a 14.7 pp lower probability of having a stunted child compared to those with inadequate assets and land ownership. The significant associative effect could be attributed to multiple economic and agency pathways. The first pathway is the wealth effects, where asset ownership could have relaxed the liquidity constraints on the availability and stability of nutritious foods. The second pathway could be an agency pathway, in that women with adequate land ownership may have had intra-household bargaining power, allowing them to allocate resources toward child nutrition.

Multiple studies support this positive association between asset ownership and stunting. In India, Harris-Fry et al. (2020) found that the size of land owned by women improved their decision-making, contributing to dietary diversity^48^. In Ethiopia, mothers who owned a house or land were less likely to have stunted children^49^. In the case of Wassie et al. (2024), asset ownership also contributed to resilience by enabling households to buffer shocks without sacrificing diet quality. Citing Santoso et al.^50^, they explained that ownership of land and other assets possibly enhanced women’s economic security and strengthened their control over resources. These pathways may have contributed to maintaining household food security and diversity, thereby leading to improved child nutrition.

The lack of significant associations between most pro-WEAI indicators and stunting should not be read as evidence that they are unimportant for child nutrition. As shown in Fig. 2, they showed directionally protective associations. One explanation is limited statistical power for some indicators when adequacy levels are either very high or very low, which reduces variation. Another explanation could measurement and timing, since empowerment may affect child growth through intermediate behaviours such as diet quality, caregiving time, and morbidity that are not fully observed in this dataset. Empirical overlaps are also expected since several pro-WEAI indicators are conceptually related therefore their independent association can appear weak even when the broader empowerment environment remains relevant. We also checked multicollinearity using variance inflation factors, and the mean VIF was 1.24 with a maximum of 2.21 (Table S2). Therefore, collinearity is unlikely to explain the non-significant coefficients.

We find marginally significant associations between marital status and stunting. Married mothers had a 15.9% point (pp) lower probability of having a stunted child than unmarried mothers. Women in partnered households may have had access to informational/emotional support, as well as shared household/child-care tasks, as part of the “social support” resource that improved child nutrition. Lee et al. (2022) argued that child/maternal support transmitted nutrition knowledge, bolstered maternal mental health, and reduced household hunger, which in turn lowered the risk of stunting in Ethiopia, India, Peru, and Vietnam^51^. Lowery et al. (2022) similarly reported that engaging family members increases support for recommended behaviours, including child nutrition^52^.

Maternal education (schooling years) was negatively associated with child stunting. Each additional year of education was associated with a 1.3 pp decrease in the probability of child stunting. This finding aligns with the well-established link between maternal education and improved child health outcomes^53,54^. In South Africa, higher maternal education was found to be associated with better health-seeking behaviours, improved maternal nutrition, and enhanced childcare practices^55,56^, also highlighted the associative pathways of the effect of schooling on child nutrition. They argue that schooling improves information processing and use of health services, skills that facilitate navigation of providers, and role/identity shifts that make caregiving and nutrition more interactive. They identified schooling as a condition that enables the transition from nutrition information to nutrition behaviour, all of which link to better child growth.

We find a strong positive association between child age and stunting. A one-month increase in age was associated with a 0.7% point higher probability of stunting, which is consistent with well-documented post-infancy growth faltering. This finding is well-documented in longitudinal studies. Lundeen et al. (2013) found that stunting prevalence increased between ages 1 and 5 years in a study that characterized post-infancy growth patterns among children aged 1, 5, and 8 years from Ethiopia, India, Peru, and Vietnam^57^. This could be attributed to nutritional challenges associated with transitions from breastfeeding or during and after the complementary feeding period, high burden of infectious diseases among children under 5 that impair nutrient absorption and increase metabolic demands, or socio-economic constraints^57,58^ [59]. This finding suggests the need for interventions targeting the period during and after the complementary feeding window, with a focus on hygiene and infection control, rather than exclusively focusing on infancy.

The findings of this study have multiple program and practical implications. First, the findings reveal that programs should not treat women’s empowerment as sufficient for child nutrition. Consequently, nutrition interventions need to pair women’s agency-building components with material and care-enabling conditions to address other structural barriers that impede child-care and feeding. Practically, the negative association between land and asset ownership and stunting suggests the need for integrated approaches that strengthen women’s resource rights and economic security. The positive association between self-efficacy and stunting suggests that gender-responsive nutrition programs need to create the needed conditions that convert that agency into child nutrition gains. For instance, improving access to quality health and nutrition services could enable women to confidently act on nutrition knowledge and preferences for improved child nutrition.

The strength of this study is its contribution to the growing of the role women’s empowerment in addressing child undernutrition. However, the analysis is associative and cannot establish causality. The potential confounding (e.g., economic conditions, program exposure, household and social conditions) and measurement issues (e.g., self-reported decision-making, reverse causality is plausible for self-efficacy), may have biased the association between women’s empowerment and stunting. Future research could use longitudinal and quasi-experimental and randomized designs the impact of gender-responsive nutrition interventions on malnutrition.

##  Conclusion

The study examined the association between women’s empowerment and child stunting in three rural districts of Malawi. We find that stunting is positively associated with child age and negatively associated with maternal schooling and being married. We also find that empowerment is multidimensional in its association with child nutrition: adequacy in ownership of land and other assets is inversely associated with stunting, while self-efficacy is positively associated, and the remaining empowerment indicators are not statistically significant although most are directionally protective. These findings hold significant relevance for Malawi, where child stunting remains a substantial public health concern. In conclusion, gender-responsive nutrition interventions need to pair women’s agency-building components with material and care-enabling conditions to address other structural barriers to child and women nutrition. Improving women’s access to quality health and nutrition services and information could enable women to translate their agency and nutrition knowledge into better feeding, hygiene, and childcare practices that support child growth.

## Supplementary Information

Below is the link to the electronic supplementary material.

Supplementary Material 1

## Data Availability

The datasets generated during and/or analysed during the current study will be stored in the CGIAR Dataverse. It will only be made available upon request to the corresponding author.
