# Supplementary material for: The association between women’s empowerment in agriculture and child stunting in Malawi
Source: Sci Rep. 2026 Feb 22;16:10183. doi: 10.1038/s41598-026-40495-6 (PMC13022126; doi:10.1038/s41598-026-40495-6)
Supplement: Supplementary file 1 — Supplementary Material 1 [file 41598_2026_40495_MOESM1_ESM.docx]

Table S1. Variables used in the logistic regression models of child stunting

| Variable block | Variable | Measurement and coding | Why included in the empowerment–nutrition framework |
| --- | --- | --- | --- |
| Outcome | Child stunting | Binary: 1=stunted, 0=not stunted, defined using HAZ < −2 SD from the WHO reference median | Captures chronic undernutrition as the focal child outcome in the empowerment–nutrition linkage |
| Pro-WEAI indicators) | Self-efficacy | Binary: 1=adequate if “agree” or greater on average across the self-efficacy items, 0=otherwise | Intrinsic agency, expected to shape caregiving confidence and follow-through on nutrition and health practices |
|  | Autonomy in income | Binary: 1=adequate if more motivated by own values than by coercion or fear of others’ disapproval | Intrinsic agency, reflects internal freedom to act on nutrition and care preferences |
|  | Attitudes about intimate partner violence | Binary: 1=adequate if husband is not justified to hit wife under any of five circumstances | Intrinsic agency, proxy for gender norms that can condition bargaining, safety, and care decisions |
|  | Input in productive decisions | Binary: 1=adequate if woman makes or feels she can make decisions in all productive activities she participates in | Instrumental agency, expected to influence production choices and food availability and diversity |
|  | Ownership of land and other assets | Binary: 1=adequate if woman owns land or at least 3 listed agricultural assets | Instrumental agency, captures resource rights and economic security that may relax food and care constraints |
|  | Access to and decisions on financial services | Binary: 1=adequate if woman decides to borrow or use credit, or has access if needed, or has a financial account | Instrumental agency, reflects liquidity and ability to respond to child health and food needs |
|  | Control over use of income | Binary: 1=adequate if woman has input on use of agricultural outputs and non-farm income for activities she is involved in | Instrumental agency, links empowerment to household allocation toward food, health, and care |
|  | Work balance | Binary: 1=adequate if works <10.5 hours/day (workload definition per pro-WEAI) | Instrumental agency constraint, reflects time trade-offs between production and caregiving |
|  | Visiting important locations | Binary: 1=adequate if visits at least 2 of 3 places weekly, or hospital/public gathering at least monthly | Instrumental agency, captures mobility and service access pathways relevant for child health and nutrition |
|  | Group membership | Binary: 1=adequate if active member in at least one community group | Collective agency, captures social capital and information channels relevant for nutrition and care |
| Child controls | Child age (months) | Continuous (months) | Captures biological and feeding-transition risk over the early life course |
|  | Child sex (female) | Binary: 1=female, 0=male | Controls for sex-differentiated growth and care patterns |
| Maternal and household controls | Mother’s age (years) | Continuous (years) | Controls for maternal life-cycle factors that may shape caregiving capacity and empowerment |
|  | Mother married | Binary: 1=married, 0=otherwise | Captures partnership and support context that can affect resources, time, and childcare |
|  | Mother’s years of schooling | Continuous (years) | Human capital pathway affecting health knowledge, service navigation, and care practices |
|  | Farming as mother’s main occupation | Binary: 1=farming, 0=otherwise | Controls for livelihood context and time allocation patterns that may confound empowerment–nutrition links |
|  | Household type: female-adult-only | Binary: 1=female-adult-only household, 0=dual-adult household, defined as households without a male decision maker present | Controls for household structure, which affects bargaining, labor, and resource access |
| Location controls | District dummies | Dedza and Mzimba South indicator variables, with Balaka as reference category | Controls for district-level heterogeneity in agro-ecology and context that may jointly shape empowerment and nutrition |

Pro-WEAI Source: <https://weai.ifpri.info/files/2023/08/Pro-WEAI_main-module_01.03.2022_online_core-only.pdf>

Table S2: VIF

Variable | VIF 1/VIF

-------------+----------------------

hh_type | 2.21 0.452901

married | 2.11 0.473592

feelinputd~r | 1.37 0.730457

incomecont~l | 1.34 0.743863

credit_acc~c | 1.22 0.821555

groupmember | 1.22 0.822952

b05 | 1.15 0.867999

district | 1.15 0.870475

selfeff | 1.11 0.903946

b11 | 1.10 0.906693

assetowner~p | 1.06 0.942315

age_mnths | 1.06 0.944786

work_balance | 1.05 0.951522

mobility | 1.04 0.958776

farming | 1.04 0.961345

autonomy_inc | 1.04 0.964351

sex | 1.03 0.973921

never_viol~e | 1.02 0.976506

-------------+----------------------

Mean VIF | 1.24
